# PTSD and post-traumatic growth among healthcare workers during COVID-19

**DOI:** 10.1192/j.eurpsy.2023.490

**Published:** 2023-07-19

**Authors:** C. Gesi, G. Cirnigliaro, R. Cafaro, M. Cerioli, F. Achilli, M. Boscacci, B. Dell’Osso

**Affiliations:** Mental health and addiction, ASST Fatebenefratelli-Sacco, Milan, Italy

## Abstract

**Introduction:**

The COVID-19 pandemic has strongly impacted mental health outcomes of healthcare workers (HWs). In spite of the large literature reporting on Post-Traumatic Stress Disorder (PTSD) symptoms, only a few studies focused on potential positive aspects that may follow the exposure to the COVID-19 pandemic, namely post-traumatic growth (PTG) among HWs.

**Objectives:**

In a large sample of Italian HWs, we aimed to investigate the prevalence of PTSD, its correlates and whether PTG dimensions independently affect the risk of PTSD during the first COVID-19 wave.

**Methods:**

An online self-report survey was submitted to HWs throughout physicians’ and nurses’ associations, social networks and researchers’ direct contacts, between April 4th and May 13th, 2020. Sociodemographic data, information about possible COVID-19 related stressful events, Impact of Event Scale-Revised (IES-R) and PTG Inventory-Short Form (PTGI-SF) scores were collected. IES-R and PTGI-SF scores were compared between subjects based on main sociodemographic, work- and COVID-19-related variables using the Student T-test or the one-way ANOVA where appropriate. Post-hoc comparisons were conducted using the Tukey test. Participants with total IES-R score >32 were assigned a provisional PTSD diagnosis and binary logistic regression analysis was conducted to investigate the contribution of each variable to the provisional PTSD diagnosis.

**Results:**

Out of 930 respondents, 256 (27,1%) reported a provisional PTSD diagnosis. Female sex (p<.001), separation from cohabiting family (p<.001), family members infected with (p<.05) or deceased due to (p<.05) COVID-19, increased workload (p<.05), relocation to a different work unit (p<.05) and unusual exposure to sufferance (p<.001) were significantly associated with higher IES-R mean scores. The median PTGI-SF score was 24. Factors associated with greater mean PTGI-SF scores were female gender (p<.001), being a nurse (p<.05), being older than 40 years (p<.05), and increased workload (p<.05). The logistic regression model showed that previous mental disorders (OR=1.65; 95% CI= 1.06-2.57) working in medical (OR=2.20; 95% CI=1.02-4.75), or service units (OR=2.34; 95% CI=1.10-4.98) (compared to frontline unit), relocation to a COVID-19 unit (OR=1.90; 95% CI=1.06-3.36), unusual exposure to sufferance (OR=2.83; 95% CI=1.79-4.48) and exposure to a traumatic event implying threat to self (compared to other work-related events) (OR=2.07; 95% CI=1.10, 3.89) significantly increase the risk of receiving a provisional diagnosis of PTSD, while the availability of personal protective equipment (OR=.61; 95% CI=.40-.94) and moderate or greater scores on PTGI-SF, particularly in the spiritual change domain (OR=.552; 95% CI=.35-.85), were found to be protective factors in relation to the PTSD diagnosis.

**Conclusions:**

Our results shed light on possible protective factors against PTSD symptoms in HWs facing COVID-19 pandemic.

**Disclosure of Interest:**

None Declared

